# Quality of patient-reported outcome measures for acute bronchitis: a systematic review of instruments and measurement properties

**DOI:** 10.1186/s41687-025-00921-1

**Published:** 2025-07-17

**Authors:** Theresa Donhauser, Katharina Piontek, Ann-Kristin Baalmann, Christian Apfelbacher

**Affiliations:** Institute of Social Medicine and Health Systems Research, Medical Faculty Magdeburg, Otto-von-Guericke University Magdeburg, Leipziger Str. 44, 39120 Magdeburg, Germany

**Keywords:** Acute bronchitis, Acute cough, Common cold, (lower) Respiratory tract infection, Patient-reported outcome measures, Measurement properties, COSMIN

## Abstract

**Background:**

Patient-reported outcome measures (PROMs) are standardized questionnaires for the assessment of health outcomes directly from the patient. A systematic evaluation of the quality of PROMs for acute bronchitis (AB) and acute cough due to (lower) respiratory tract infection or common cold has not yet been performed. The present study aimed to systematically review the quality of available PROMs for AB and acute cough due to (lower) respiratory tract infection or common cold for use in adults and children.

**Methodology:**

Embase, PubMed and Web of Science were searched for studies reporting on the development and/or validation of any PROMs for AB and acute cough due to (lower) respiratory tract infection or common cold. We assessed the methodological quality of each included study, evaluated the quality of measurement properties per PROM and study, and graded the evidence according to the COnsensus-based Standards for the selection of health Measurement INstruments (COSMIN) methodology. Based on the overall evidence, we derived recommendations for use of the instruments.

**Results:**

We included three studies on three PROMs for adults measuring disease severity (Acute Bronchitis Severity Score (ABSS); Symptom Diary) and cough-related quality of life (Leicester Cough Questionnaire (LCQ-acute)). For children, we included two studies on two PROMs assessing quality of life (Parent-proxy Children’s Acute Cough-specific QoL Questionnaire (PAC-QoL_16_) and its Short Form (PAC-QoL_6_)), and one study on a PROM assessing cold symptoms (Child Cold Symptom Questionnaire (CCSQ)). All instruments were classified as COSMIN category B except for the PAC-QoL_6_, indicating that they have the potential to be recommended, but require further validation. The PAC-QoL_6_ cannot be recommended for use (COSMIN category C). Content validity is a shortcoming of all identified PROMs.

**Conclusions:**

None of the identified PROMs can be unrestrictedly recommended for use in future research. For adults, the LCQ-acute appears the most suitable tool warranting further validation. Given the intensive work on scale development and testing for PROM design, the CCSQ is promising for use in children. Content validity assessments involving patients and experts are highly recommended for all identified PROMs.

**Systematic review registration:**

OSF (10.17605/OSF.IO/3G6CP).

**Supplementary Information:**

The online version contains supplementary material available at 10.1186/s41687-025-00921-1.

## Background

Acute cough, a major symptom of acute bronchitis (AB) and common cold [1], is related to impairment of the individual’s daily activities and health-related quality of life (HRQoL) [2]. Given the self-limiting nature of the condition, treatment is aimed at symptom alleviation [3], and over-the-counter medications are primarily used [4]. For promoting patient-centric care, the patient’s perspective is crucial when evaluating the effectiveness of these treatments. For this purpose, patient-reported outcome measures (PROMs) are suitable tools [5]. PROMs are standardized questionnaires for the assessment of various health outcomes directly from the patient including disease symptoms or treatment side effects, functional outcomes, and multidimensional constructs such as HRQoL [6].

In research and clinical practice, it is a substantial challenge to select a reliable and valid tool from the multitude of PROMs available. In addition to considerations related to the construct to be measured and the target population, it is necessary to evaluate the quality of the measurement properties of available instruments. To facilitate the selection of PROMs, the COnsensus-based Standards for the selection of health Measurement INstruments (COSMIN) methodology [7] has been introduced. The COSMIN methodology provides a methodological approach including detailed, standardized and transparent criteria, and practical tools for selecting the most appropriate instrument [8]. A systematic assessment of existing PROMs for AB and acute cough due to (lower) respiratory tract infection or common cold and an evaluation of the methodological quality of these instruments using the COSMIN methodology has not yet been undertaken.

The aim of the present study was to conduct a systematic review of the quality of disease-specific PROMs for AB and acute cough due to (lower) respiratory tract infection or common cold for use in adults and children. Concretely, we aimed

1. To systematically assess the measurement properties of existing disease-specific PROMs for AB and acute cough due to (lower) respiratory tract infection or common cold, i.e.,

i. To evaluate the quality of development and/or validation studies
ii. To evaluate the psychometric properties of the identified PROMs including aspects of interpretability and feasibility
iii. To grade the evidence

2. To derive recommendations for the use of the identified PROMs in future research

## Methods

### Protocol and registration

The methods of this systematic review were developed based on the recommendations of the Preferred Reporting Items for Systematic Reviews and Meta-Analyses Protocols (PRISMA-P) statement [9] and the COSMIN guideline and manual for systematic reviews of PROMs [7, 10]. The corresponding study protocol was registered in the open registries network (OSF; 10.17605/OSF.IO/3G6CP).

### Literature search

A systematic literature search was performed on 21 September 2023 in the databases PubMed, Web of Science and Embase. The search strategy included the following elements:

A. Target population: Adults and children with AB or acute cough due to lower respiratory tract infection or common cold (in the following refered to as “acute cough” for easier readability). To allow for a broad sensitivity, a comprehensive compilation of controlled vocabulary and free text terms based on the literature were applied.
B. Construct of interest: All PROMs related to AB and acute cough in adults and children were included.
C. Measurement properties: The validated and sensitive search filter developed by Terwee et al. [11] for PubMed was used and adapted for the search in Web of Science and Embase.
D. Feasibility: The search strategy for this element was based on the search terms for the concept ‘feasibility’ of Heinl et al. [12].
E. Individual PROMs: A list of existing PROMs in the context of AB and acute cough already known were included.
F. Exclusion filter: The filter by Terwee et al. [11] was used to exclude irrelevant publication types.

The search elements were combined as follows for its application in PubMed: (((A AND B AND (C OR D)) OR (C AND E)) NOT F); in words: (((population AND construct AND (measurement properties OR feasibility)) OR (individual PROMs AND measurement properties)) NOT (exclusion filter)). For search in Web of Science and Embase, the search strategy for PubMed was adapted choosing appropriate syntax and index terms. In addition, the reference lists of the included studies were screened for further relevant studies.

There were no restrictions regarding publication date and language. All records were exported to Citavi 6 and subsequently exported to Rayyan [13] for further processing (i.e., title/abstract and full text screening). An update of our literature search was conducted on 12 July 2024.

### Eligible studies

Inclusion and exclusion criteria are displayed in Table 1. Eligible studies concerned any PROMs for adults and children with AB or acute cough due to respiratory tract infection or common cold, and at least 50% of the study sample needed to consist of individuals fulfilling these criteria. The development and/or validation of a PROM needed to be the principal aim of selected studies. Studies only using the PROM as an outcome measure and studies in which the PROM was used for the validation of another instrument were excluded. Only full-text articles were included because abstracts often provide very limited information on the design of a study.

**Table 1:** Inclusion and exclusion criteria

	Inclusion criteria	Exclusion criteria
Population	Adults and children with acute bronchitis/acute cough due to lower respiratory tract infection or common cold	Chronic bronchitis and other diseases of the lower respiratory tract
Study design	PROM development and/or validation study	All other study designs
Outcome	All patient-reported and proxy-reported outcomes	Non patient-reported outcomes, e.g., biomarkers, laboratory data
Type of measurement instrument	Patient- and proxy-reported outcome measurement instruments	All others
Publication type	Articles with available full text	Abstracts and other publication types

*Abbreviations.* PROM = patient-reported outcome measure

### Study selection

After the list of records was created and duplicates were removed, titles and abstracts were evaluated by two independent reviewers according to the inclusion and exclusion criteria. Full-text articles for publications considered eligible at this stage were searched and also evaluated by two reviewers independently regarding eligibility. If any disagreement occurred, consensus was reached by consulting a third reviewer.

### Data extraction

The measurement properties of the single PROMs were assessed in the following order:

1. Evaluation of content validity
2. Evaluation of internal structure including structural validity, internal consistency, and cross-cultural validity/measurement invariance
3. Evaluation of remaining measurement properties including reliability, measurement error, criterion validity, hypotheses testing for construct validity, and responsiveness

#### Quality of development and validation studies

The methodological quality of each study on a measurement property was judged by two independent reviewers using the COSMIN Risk of Bias checklist [10]. This checklist consists of 10 boxes including all standards needed to assess the quality of a study on that specific measurement property: PROM development, content validity, structural validity, internal consistency, cross-cultural validity/measurement invariance, reliability, measurement error, criterion validity, hypotheses testing for construct validity, and responsiveness. The methodological quality of each study on a specific measurement property was evaluated on a 4-point rating scale as either very good, adequate, doubtful or inadequate. The overall quality of a study was determined by the lowest rating of any standard in the box (“worst score counts principle”).

Additionally, relevant data on characteristics of the included PROMs and study populations, and data on interpretability and feasibility of the instruments was extracted.

#### *Quality of* measurement *properties*

The result of each single study on a measurement property was rated against the criteria for good measurement properties [7]. Measurement properties were rated as either sufficient (+), insufficient (-), or indeterminate (?).

#### *Grading of* the *evidence*

The quality of the evidence was summarized per measurement property per PROM, and the summarized results were also rated against the criteria for good measurement properties [7]. Using the Grading of Recommendations Assessment, Development and Evaluation (GRADE) approach, the quality of evidence was graded considering the methodological quality of studies, total sample size, and consistency of results [14]. The quality of evidence was rated as either high, moderate, low, or very low.

#### Generating recommendations for use of the identified PROMs

Each PROM was assigned to a recommendation category according to its methodological quality as follows [7]:

A. PROMs with evidence for sufficient content validity (any level) and at least low-quality evidence for sufficient internal consistency.
B. PROMs categorized not in A or C.
C. PROMs with high-quality evidence for an insufficient measurement property.

Results obtained from PROMs of category A are considered trustworthy, and these instruments can be recommended for use. PROMs of category B have the potential to be recommended for use, but further validation is needed. PROMs of category C cannot be recommended for use. If only PROMs of category B are available, the PROM with the best evidence for content validity can be preliminarily recommended for use until further evidence is given [14].

## Results

### Literature search and eligible studies

**Fig. 1:**
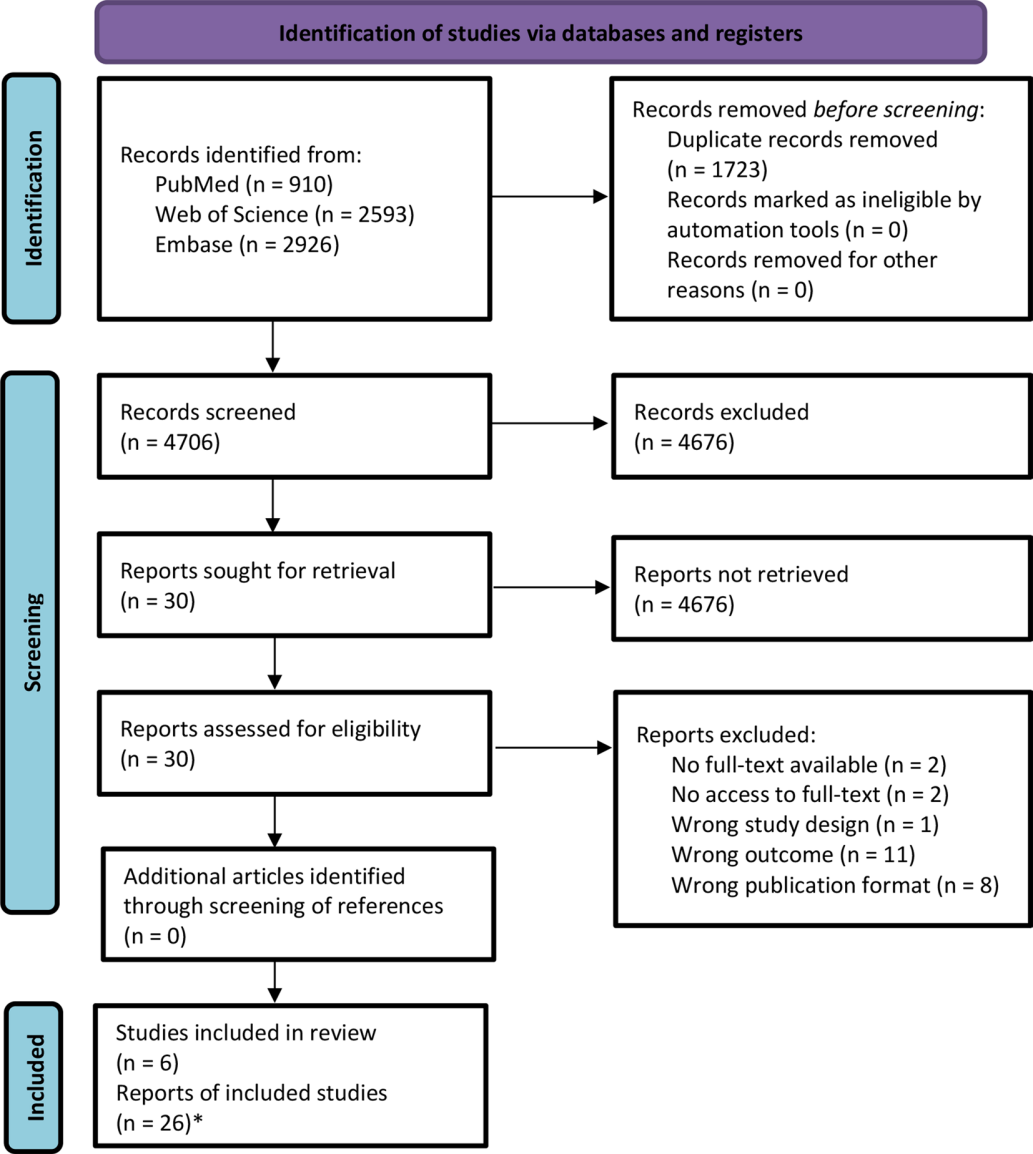
Adapted Preferred Reporting Items for Systematic Reviews and Meta-Analyses (PRISMA) Protocols 2009 [9] flow diagram. *Number of single studies on measurement properties in the included studies

The literature search yielded 6,429 records, of which 4,706 were screened for eligibility after deduplication (Fig. 1). In total, six studies were included for data extraction based on the results of the full-text screening. No further relevant studies were identified in the reference lists of the included studies. One study each reported on the following PROMs for adults: Acute Bronchitis Severity Score (ABSS) [15], Leicester Cough Questionnaire (LCQ-acute) [16], and Symptom Diary [17]. For children, one study reported on the Parent-proxy Children’s Acute Cough-specific Quality of Life Questionnaire (PAC-QoL_16_) [18], one study reported on its Short Form (PAC-QoL_6_) [19], and one study reported on the Child Cold Symptom Questionnaire (CCSQ) [20].

The update of our literature search did not yield new eligible studies.

### Data extraction

#### Characteristics of the included PROMs and study populations

An overview of the characteristics of the included PROMs and study populations can be found in the online material 1 and 2.

#### Evaluation of PROM development and content validity studies

The results of the ratings of the development and content validity studies of PROMs in adults and children are displayed in Tables 2 and 3, respectively. Concerning instruments for use in adults, PROM development of the ABSS [15] and the Symptom Diary [17] were rated as “inadequate” due to a lack of patient involvement in the design of the instruments. The development of the LCQ-acute [16] also received an “inadequate” rating since the original PROM (LCQ) was initially developed for chronic cough. For the ABSS and the Symptom Diary, no content validity studies were available, and content validity of these instrument was therefore solely rated by the reviewers. For the LCQ-acute, one content validity study was available [16], which was rated as “doubtful” since the study procedure was not adequately described. The reviewers rated the content validity of the ABSS and the Symptom Diary as sufficient. Along with inadequate instrument development, this evaluation resulted in very low quality of evidence for content validity of both instruments. Considering the data reported in the available study, the content validity of the LCQ-acute was rated as sufficient. Since the development of the instrument was rated as “doubtful”, this evaluation resulted in moderate quality of evidence for content validity.

With regard to PROMs for use in children, the development of the PAC-QoL_16_ [18] and PAC-QoL_6_ [19] was rated as “inadequate” due to the lack of a cognitive interview study and/or pilot test. The development of the CCSQ [20] was rated as “doubtful” since patients were not asked about comprehensiveness in the cognitive interview study. For all three instruments, no content validity studies were available. Evaluation of the PAC-QoL_16_ [18] resulted in an indeterminate content validity rating since the reviewers found the questions of the instrument to be focused on the quality of life of the parents of the sick children and not on the quality of life of the children themselves. Since no copy of the PAC-QoL_6_ [19] was available to the reviewers, content validity could not be rated and remains therefore indetermined. Reviewers rated the content validity of the CCSQ [20] as sufficient, with very low quality of evidence. Since no high quality evidence for an insufficient content validity of any PROM was found, the quality of the remaining measurement was evaluated.

**Table 2:** Content validity rating of the included instruments for adults

		PROM development	Content validity	Rating of Reviewers	Overall rating per PROM
ABSS	*Methodological quality of the study*	Inadequate	NA		
	Overall rating*	NA, due to inadequate methodological quality		Relevance: + Comprehensiveness: + Comprehensibility: NA Content validity rating: +	**Relevance: + Comprehensiveness: + Comprehensibility: ± Content validity rating: +**
	*Quality of evidence*			Very low (due to Risk of Bias)	
LCQ-acute	*Methodological quality of the study*	Inadequate	Doubtful		
	Overall rating*	NA, due to inadequate methodological quality	Relevance: + Comprehensiveness: + Comprehensibility:? Content validity rating: +	Relevance: + Comprehensiveness: + Comprehensibility: NA Content validity rating: +	**Relevance: + Comprehensiveness: + Comprehensibility: + Content validity rating: +**
	*Quality of evidence*		Moderate (due to Risk of Bias)		
Symptom Diary	*Methodological quality of the study*	Inadequate	NA		
	Overall rating*	NA, due to inadequate methodological quality		Relevance: + Comprehensiveness: + Comprehensibility: NA Content validity rating: +	**Relevance: + Comprehensiveness: + Comprehensibility: + Content validity rating: +**
	*Quality of evidence*			Very low (due to Risk of Bias)	

*Abbreviations.* ABSS = Acute Bronchitis Severity Score, LCQ-acute = Leicester Cough Questionnaire, NA = not assessed, PROM = patient-reported outcome measure

*(+) sufficient, (-) insufficient, (?) indeterminate, (±) inconsistent

**Table 3:** Content validity rating of the included instruments for children

		PROM development	Content validity	Rating of Reviewers	Overall rating per PROM
PAC-QoL_16_	*Methodological quality of the study*	Inadequate	NA		
	Overall rating*	NA, due to inadequate methodological quality		Relevance:? Comprehensiveness:? Comprehensibility: NA Content validity rating:?	**Relevance:? Comprehensiveness:? Comprehensibility: + Content validity rating:?**
	*Quality of evidence*			No grading if overall rating is indeterminate	
PAC-QoL_6_	*Methodological quality of the study*	Inadequate			
	Overall rating*	NA, due to inadequate methodological quality	NA	NA	**?**
	*Quality of evidence*				No grading if overall rating is indeterminate
CCSQ	*Methodological quality of the study*	Doubtful			
	Overall rating*	Relevance: + Comprehensiveness:? Comprehensibility: + Content validity rating: +	NA	Relevance: + Comprehensiveness: + Comprehensibility: NA Content validity rating: +	**Relevance: + Comprehensiveness: + Comprehensibility: + Content validity rating: +**
	*Quality of evidence*	Low (due to Risk of Bias)			

*Abbreviations.* CCSQ = Child Cold Symptom Questionnaire, NA = not assessed, PAC-QoL_16_ = Parent-proxy Children’s Acute Cough-specific Quality of Life Questionnaire, PAC-QoL_6_ = Parent-proxy Children’s Acute Cough-specific Quality of Life Questionnaire – Short Form, PROM = patient-reported outcome measure

*(+) sufficient, (-) insufficient, (?) indeterminate, (±) inconsistent

#### Evaluation of remaining measurement properties

In total, the methodological quality of 26 measurement properties was assessed (Table 4). Among those, nine reached a “very good” rating (34.6%), eight reached an “adequate” rating (30.8%), five reached a “doubtful” rating (19.2%), and four reached an “inadequate” rating (15.4%).

**Table 4:** Evaluation of the remaining measurement properties

	Methodological quality (rating^a, b^)						
	Structural validity	Internal consistency		Test-Retest Reliability	Construct validity (comparator instrument)	Construct validity (known-groups)	Responsiveness
**PROMs for adults**							
ABSS	Adequate (?)	Doubtful (?)	2x Inadequate (?)			Adequate (+)	
LCQ-acute		Doubtful (?)	Inadequate (?)		Adequate (+)		Very good (±) Adequate (+)
Symptom Diary					Adequate (±)		Adequate (-)
**PROMs for children**							
PAC-QoL_16_		2x Doubtful (?)	Very good (+), Very good (-)		2x Very good (±)	2x Adequate (+)	2x Very good (+)
PAC-QoL_6_		Doubtful (?)	Very good (-), Inadequate (-)		Very good (±)		
CCSQ	NA						

*Abbreviations.* ABSS = Acute Bronchitis Severity Score, CCSQ = Child Cold Symptom Questionnaire, LCQ-acute = Leicester Cough Questionnaire, NA = not assessed, PAC-QoL_16_ = Parent-proxy Children’s Acute Cough-specific Quality of Life Questionnaire, PAC-QoL_6_ = Parent-proxy Children’s Acute Cough-specific Quality of Life Questionnaire – Short Form, PROM = patient-reported outcome measure

^a^(+) sufficient, (-) insufficient, (?) indeterminate, (±) inconsistent

^b^No study has analyzed cross-cultural validity/measurement invariance, measurement error and criterion validity

#### Details on interpretability and feasibility of the included PROMs

Regarding interpretability of the PROMs for adults, a minimal important difference (MID) of 2.5 was suggested for the LCQ-acute [16]. For the ABSS, floor and ceiling effects of 1.8% and 0.2%, respectively, were reported [15]. With respect to the PROMs for children, MIDs of 0.98 [18] and of 0.71–1.02 [19] were suggested for the PAC-QoL_16_. For its Short Form, PAC-QoL_6_, a MID ranging from 0.73 to 1.11 was proposed [19].

Regarding feasibility, all questionnaires with the exception of the PAC-QoL_16_ and PAC-QoL_6_ are self-reported. Scoring of the PAC-QoL_16_, the PAC-QoL_6_ and the LCQ-acute is performed by addition of the scores of the single items. Completion time of the questionnaire was stated for none of the PROMs. Furthermore, the PAC-QoL_6_ was not available upon request from the authors. Only for the CCSQ, a copyright statement was included in the assessed article [20].

#### Summary of findings and recommendation

The summary of findings on measurement properties of the assessed PROMs is depicted in Table 5. The results are presented separately for all existing subscales, if available, even in the absence of evidence for sufficient structural validity, to maximize the accuracy of our overall rating of measurement properties. Further, the results for construct validity of the PAC-QoL_16_ and the PAC-QoL_6_ are displayed separately for both measurement points used in the respective studies.

**Table 5:** Summary of findings

	Summary or pooled result	Overall rating	Quality of evidence
**PROMs for adults**			
**ABSS**			
Structural validity	Not all information for a sufficient rating reported, *n* = 649	Indeterminate	-
Internal consistency	Alpha = 0.66; no evidence for sufficient structural validity, *n* = 649	Indeterminate	-
Test-Retest Reliability	No ICCs reported, *n* = 530–649	Indeterminate	-
Construct validity (Known-groups)	3 out of 3 hypotheses confirmed, *n* = 501–606	Sufficient	Moderate (due to Risk of Bias)
**LCQ-acute**			
Internal consistency	Alpha = 0.94, no evidence for sufficient structural validity, *n* = 30	Indeterminate	-
Test-Retest Reliability	Not all information for a sufficient rating reported, *n* = 6	Indeterminate	
Construct validity (comparator instrument)	1 out of 1 hypothesis confirmed, *n* = 30	Sufficient	Low (due to Risk of Bias and imprecision)
Responsiveness	**Total score and physical subdomain**: hypothesis confirmed **Psychological and social subdomain**: hypothesis not confirmed 3 out of 5 hypotheses confirmed, *n* = 30	**Total score/physical subdomain**: sufficient **Psychological/social subdomain**: insufficient	Low (due to imprecision)
**Symptom Diary**			
Construct validity (comparator instrument)	10 out of 14 hypotheses confirmed, *n* = 88	Inconsistent (inconsistency could not be resolved)	-
Responsiveness	1 out of 7 hypotheses confirmed, *n* = 88	Insufficient	Low (due to Risk of Bias and imprecision)
**PROMs for children**			
**PAC-QoL**_**16**_			
Internal consistency	**Total score**: Alpha = 0.94–0.95, no evidence for sufficient structural validity, *n* = 106–238	Indeterminate	-
Test-Retest Reliability	ICCs = 0.61–0.75, *n* = 186	Inconsistent (inconsistency could not be resolved)	-
Construct validity (comparator instrument)	*Enrollment* **Total score**: 17 out of 34 hypotheses confirmed (*physical subdomain*: 10/34 hypotheses confirmed, *social subdomain*: 14/34 hypotheses confirmed, *emotional subdomain*: 13/34 hypotheses confirmed) *Day 3* **Total score**: 16 out of 34 hypotheses confirmed (*physical subdomain*: 11/34 hypotheses confirmed, *social subdomain*: 23/34 hypotheses confirmed, *emotional subdomain*: 18/34 hypotheses confirmed), *n* = 142–238	Inconsistent (inconsistency could not be resolved)	
Construct validity (Known-groups)	2 out of 2 hypotheses confirmed, *n* = 152	Sufficient	High
Responsiveness	2 out of 2 hypotheses confirmed, *n* = 152	Sufficient	High
**PAC-QoL**_**6**_			
Internal consistency	Alpha = 0.84–0.87, no evidence for sufficient structural validity, *n* = 332	Indeterminate	
Test-Retest Reliability	ICCs = 0.63–0.68, *n* = 332	Insufficient	High
Construct validity (comparator instrument)	*Enrollment* 10 out of 22 hypotheses confirmed *Day 3* 17/22 hypotheses confirmed, *n* = 56–142	*Enrollment* Inconsistent (inconsistency could not be resolved) *Day 3* Sufficient	High
**CCSQ**			
Other measurement properties not assessed			

*Abbreviations.* ABSS = Acute Bronchitis Severity Score, CCSQ = Child Cold Symptom Questionnaire, ICC = intra-class correlation coefficient, LCQ-acute = Leicester Cough Questionnaire, n = sample size, PAC-QoL_16_ = Parent-proxy Children’s Acute Cough-specific Quality of Life Questionnaire, PAC-QoL_6_ = Parent-proxy Children’s Acute Cough-specific Quality of Life Questionnaire – Short Form, PROM = patient-reported outcome measure

Recommendations for future use of the identified PROMs are displayed in Table 6.

**Table 6:** Recommendations for use of the identified instruments

	Category A		Category C	
PROM	Sufficient content validity (any level of evidence)	At least low quality evidence for sufficient internal consistency	High quality evidence for an insufficient measurement property	Recommendation
**PROMs for adults**				
ABSS	✓	×	×	B
LCQ-acute	✓	×	×	B
Symptom Diary	✓	×	×	B
**PROMs for children**				
PAC-QoL_16_	×	×	×	B
PAC-QoL_6_	×	×	✓	C
CCSQ	✓	×	×	B

*Abbreviations.* ABSS = Acute Bronchitis Severity Score, CCSQ = Child Cold Symptom Questionnaire, LCQ-acute = Leicester Cough Questionnaire, PAC-QoL_16_ = Parent-proxy Children’s Acute Cough-specific Quality of Life Questionnaire, PAC-QoL_6_ = Parent-proxy Children’s Acute Cough-specific Quality of Life Questionnaire – Short Form, PROM = patient-reported outcome measure

## Discussion

In the present systematic review on the quality of PROMs for AB and acute cough due to (lower) respiratory tract infection or common cold following the COSMIN methodology, we identified three instruments for use in adults (ABSS, Symptom Diary, LCQ-acute) and three instruments for use in children (PAC-QoL_16,_ PAC-QoL_6,_ CCSQ). All instruments except for the PAC-QoL_6_ were classified as COSMIN category B, indicating that they have the potential to be recommended for use in future research, but require further validation. The PAC-QoL_6_ cannot be recommended for use (COSMIN category C). The included PROMs have substantial conceptual and methodological weaknesses, in particular with regard to PROM development and content validity, but also data on other important measurement properties such as structural validity are lacking.

Content validity is considered the most important measurement property [21], and PROM development guidelines strongly recommend patient involvement in several stages from concept elicitation and item generation to content validity assessments in cognitive interviews to capture relevance, comprehensiveness and comprehensibility of a questionnaire from the patients’ perspective. Reflecting the growing importance of patients’ voices in the health care system, patient involvement in PROM development is also required by regulatory authorities such as the European Medicines Agency (EMA) and the U.S. Food and Drug Administration (FDA) in the drug approval process [22]. Not meeting these requirements, ABSS and Symptom Diary assessing symptom severity in adults with AB were developed based on research literature and expert opinion. Further, no content validity studies were available, and the present content validity assessments are based solely on the reviewers’ rating, which strongly limits the quality of evidence. Moreover, data on structural validity, which is a critical measurement property for a recommendation of a PROM according to the COSMIN methodology, are not available. Initially developed for use in adults with chronic bronchitis, the LCQ-acute measuring cough-related quality of life has been adapted and validated in a population of adults with acute cough. While we found sufficient content validity of the instrument in the target population based on the available content validity study, also for this instrument evidence for structural validity is lacking. Reflecting weaknesses of the validation study, test-retest-reliability could not be determined since only few patients had been stable over the two-week study period, indicating that this time interval is too long to assess outcomes of acute cough. However, to facilitate the application of the instrument, data on MID are available for the LCQ-acute total score.

Regarding PROMs for children, PAC-QoL_16_ and its Short Form PAC-QoL_6_ are parent-proxy measures for the assessment of the child’s cough-specific quality of life. Parents were involved in the design of the instrument, but no content validity study was performed, and also for these tools, no data on structural validity are available. Notably, we found high quality evidence for insufficient test-retest-reliability of the PAC-QoL_6_ based on retesting at day 3, which might be related to parents’ difficulties in accurately assessing their child’s health status and well-being. Based on extensive scale development including item generation (e.g., draft tasks, illustrations of symptoms) and cognitive debriefing involving children, the CCSQ aims to assess symptoms of common cold in children aged 6 to 11 years using various recall periods corresponding to the capability of the target population. Data from content validity studies are still lacking, but in view of the profound PROM development, the CCSQ is promising for use and warrants further validation.

With respect to the application of the identified PROMs in research and clinical practice, the following aspects should be considered. Originally, PROMs were developed for use in research, which requires high-quality instruments in terms of measurement properties. Over time, the application of PROMs has extended including for example supporting clinical decision making, comparing outcomes among health-care providers, stimulating quality improvement and evaluating practices [6]. Furthermore, in the clinical context, PROMs are essential to support patient-centered care [23]. While our evaluation revealed certain limitations in the measurement quality of single instruments, which are critical for their use in clinical studies, these do not necessarily preclude the use of these instruments in the clinical setting. For example, we found insufficient responsiveness of the Symptom Diary, and indeterminate and inconsistent ratings for the ABSS and the PAC-QoL_16_, but the quality of evidence was only low. According to the COSMIN approach, this is no reason for excluding these instruments from future use in total, but indicates the necessity of future validation studies [14]. In this regard, the ABSS and the Symptom Diary may be useful for assessing patients’ symptoms and disease monitoring over time pending future validation analyses. Likewise, considering PROMs for children, proxy measures are important tools in clinical care, and the PAC-QoL_16_ may be applicable after further validity evidence is generated. Besides specific measurement properties, interpretability is a key aspect for application of PROMs in clinical practice, which can substantially improve clinical decision-making and the impact from using PROMs in clinical practice [24]. In this regard, data on MID of the LCQ-acute and the PAC-QoL_16_ may facilitate the interpretation of their scores and thus their practical applicability. In conclusion, acknowledging the intended context of use is essential, and some instruments may remain fit for purpose in clinical practice despite limitations in specific research populations.

### Strengths and limitations

This work has several strengths regarding the methodology: the protocol was pre-registered, three large databases (PubMed, Web of Science, Embase) were used, reference lists were checked and a sensitive search filter was applied, the eligibility criteria were pre-defined, and the COSMIN guidelines were followed in order to assess the methodological quality of the included studies. No additional studies were found in the reference lists or were provided by the contacted authors, which supports the quality of the search filter. Additionally, in every step of the procedure, at least two independent reviewers were involved and different opinions on certain decisions were frequently discussed and resolved by involving a third reviewer, if necessary.

One limitation may arise from the fact that for all PROMs except for the LCQ-acute no content validation studies are available. Consequently, the rating of the content validity was based on the reviewers’ rating, if the questionnaire was available to the authors. Since the authors’ rating is less accurate than a specific content validation assessment, this may have influenced the accuracy of the rating of the content validation of the existing PROMs in general.

## Conclusions

Currently none of the identified PROMs for adults and children with AB and acute cough due to (lower) respiratory tract infection or common cold can be unrestrictedly recommended for use in future research. The LCQ-acute for adults and the CCSQ for children appear to be the most suitable tools warranting further validation. Intensive content validity assessments involving patients and experts are highly recommended for all identified PROMs.

## Electronic supplementary material

Below is the link to the electronic supplementary material.

Supplementary Material 1

Supplementary Material 2

## Data Availability

Data is available from the corresponding author upon reasonable request.

## Abbreviations

AB: Acute bronchitis
ABSS: Acute Bronchitis Severity Score
CCSQ: Child Cold Symptom Questionnaire
COSMIN: COnsensus-based Standards for the selection of health Measurement INstruments
GRADE: Grading of Recommendations Assessment, Development and Evaluation
HRQoL: Health-related quality of life
LCQ-acute: Leicester Cough Questionnaire
PAC-QoL: Parent-proxy Children’s Acute Cough-specific QoL Questionnaire
PROM: Patient-reported outcome measure
