# Supplementary material for: Quality of patient-reported outcome measures for acute bronchitis: a systematic review of instruments and measurement properties
Source: J Patient Rep Outcomes. 2025 Jul 17;9:92. doi: 10.1186/s41687-025-00921-1 (PMC12271018; doi:10.1186/s41687-025-00921-1)
Supplement: Supplementary file 1 — Supplementary Material 1 [file 41687_2025_921_MOESM1_ESM.pdf]

Online Resource 1 Characteristics of the included PROMs

	Construct	Target population	Recall period	(Sub)scales (number of items)	Response options	Available translations
**PROMs for adults**						
**ABSS**	Acute bronchitis severity	Adults with acute bronchitis	24 hours	0 (11 items): Symptoms (7 items), Examination (3 items), Laboratory (gram strain; 1 item)	5-point Likert scale: 0 (very mild) to 5 (very serious)	English
**LCQ-acute**	Cough-related quality of life	Patients with acute cough	24 hours	3 (19): physical (8 items), psychological (7 items), social (4 items)	7-point Likert scale: 1 (all of the time) to 7 (none of the time)	English
**Symptom Diary**	Severity of common symptoms associated with acute LRTi	Patients with LRTi	24 hours	1 (6)	7-point Likert scale: 0 (normal/not affected) to 6 (as bad as it could be)	English
**PROMs for children**						
**PAC-QoL$_{16}$**	Acute cough-specific quality of life	(Parents of) children with acute cough	24 hours	3 (16): Physical domain (11 items), social domain (3 items), emotional domain (2 items)	7-point Likert scale: 1 (all of the time) to 7 (none of the time) // 1 (very very worried/concerned) to 7 (not worried/concerned)	English
**PAC-QoL$_6$**	Acute cough-specific quality of life	(Parents of) children with acute cough	24 hours	0 (6)	7-point Likert scale	English
**CCSQ**	Cold symptoms	Children of 6-11 years with cold symptoms	Various recall periods, e.g., „right now“, „today“, „when you woke up this morning“	1 (afternoon: 18 items, morning: 15 items)	5-point Likert scale: 0 (not at all) to 4 (a lot)	English

Abbreviations. ABSS = Acute Bronchitis Severity Score, CCSQ = Child Cold Symptom Questionnaire, LCQ-acute = Leicester Cough Questionnaire, LRTI = lower respiratory tract infection (LRTi), PAC-QoL$_{16}$ = Parent-proxy Children’s Acute Cough-specific QoL Questionnaire, PAC-QoL$_6$ = Parent-proxy Children’s Acute Cough-specific QoL Questionnaire – Short Form, PROM = patient-reported outcome measure.
