# Supplementary material for: Quality of patient-reported outcome measures for acute bronchitis: a systematic review of instruments and measurement properties
Source: J Patient Rep Outcomes. 2025 Jul 17;9:92. doi: 10.1186/s41687-025-00921-1 (PMC12271018; doi:10.1186/s41687-025-00921-1)
Supplement: Supplementary file 2 — Supplementary Material 2 [file 41687_2025_921_MOESM2_ESM.pdf]

Online resource 2 Characteristics of the included study populations

	Population		PROM administration			
	N	Age mean (*SD*)	Setting	Country	Language	Response rate
**PROMs for adults**						
**ABSS**	649	29.9 years (9.1)	Clinics/hospitals	Kenya	NR	NR
**LCQ-acute**	Content validity: 10; Psychometrics: 30	32 years (10)	NR	United Kingdom	English	NR
**Symptom Diary**	88	NR	General practices	United Kingdom	English	88%
**PROMs for children**						
**PAC-QoL$_{16}$**	155[a]; 83[b]	Median: 2.17 years (IQR = 1.25-4.58)[a]; Median: 2.04 years (IQR = 1.08–4.06)[b]	emergency department and community based health centers	Australia	English	NR
**PAC-QoL$_{6}$**	PAC-QoL cohort: 238; emergency department cohort: 94	**PAC-QoL cohort:** Median: 2.17 years (IQR = 1.21–4.21); **Emergency department cohort:** Median: 1.75 years (IQR = 0.90–3.63)	emergency department and community-based health centers	Australia	English	NR
**CCSQ**	Children: 39, Parents: 10	Children: 8.2 years, Parents: 36.7 years	research facility or in a room in a public building such as a library	United States	English	NR

Abbreviations. ABSS = Acute Bronchitis Severity Score, CCSQ = Child Cold Symptom Questionnaire, IQR = inter-quartile range, LCQ-acute = Leicester Cough Questionnaire, N = sample size, NR = not reported, PAC-QoL$_{16}$ = Parent-proxy Children's Acute Cough-specific Quality of Life Questionnaire, PAC-QoL$_{6}$ = Parent-proxy Children's Acute Cough-specific Quality of Life Questionnaire – Short Form, PROM = patient-reported outcome measure, SD = standard deviation.

[a]Anderson-James et al., 2015

[b]Anderson-James et al., 2021
